# Corrigendum: Reconstruction and Simulation of a Scaffold Model of the Cerebellar Network

**DOI:** 10.3389/fninf.2019.00051

**Published:** 2019-07-10

**Authors:** Stefano Casali, Elisa Marenzi, Chaitanya Medini, Claudia Casellato, Egidio D'Angelo

**Affiliations:** ^1^Neurophysiology Unit, Neurocomputational Laboratory, Department of Brain and Behavioral Sciences, University of Pavia, Pavia, Italy; ^2^IRCCS Mondino Foundation, Pavia, Italy

**Keywords:** cerebellum, computational spiking models, Python, pyNEST, pyNEURON, connectome

In the published article, there was an error regarding the affiliation for **Egidio D'Angelo**. As well as having affiliation(s) **1**, the author should also have **IRCCS Mondino Foundation, Pavia, Italy**. The authors apologize for this error and state that this does not change the scientific conclusions of the article in any way. The original article has been updated.

