# Supplementary material for: Reconstruction and Simulation of a Scaffold Model of the Cerebellar Network
Source: Front Neuroinform. 2019 May 15;13:37. doi: 10.3389/fninf.2019.00037 (PMC6530631; doi:10.3389/fninf.2019.00037)
Supplement: Supplementary file 2 [file Data_Sheet_1.pdf]

Supplementary Material

Reconstruction and Simulation of a Scaffold Model of the Cerebellar Network

Network architecture and reconstruction

Cell placement workflow

Given the following data:

- cell type (e.g., granule cell, *GrCs*)
- layer where the cell type can be found (e.g., granular layer, *GL*)

we know:

- (mean) radius of the *soma* of the selected cell type (e.g., 2.5 μm), ***r***
- (mean) thickness of the layer (e.g., 150 μm), ***thick***
- estimated density distribution for the cell type in a volume (e.g., 3.9 x 10^{-3}neurons/μm^3), ***dens***

In order to fill a given volume of 400 x 400 x ***thick***μm^3 with a selected cell type:

1. **the layer is partioned in sublayers**; a potential volume for each cell (including an empty surrounding volume), *v_cell*, is calculated as *1/dens*. The radius of *v_cell*, *v_cell_r*, is calculated as $\sqrt[3]{\frac{3}{4 \cdot \pi \cdot v_{cell}}}$. The number of sublayers to be created is then determined by $\frac{thick}{1.5 \cdot v_{\square}}$, which allows to determine the number of cells assigned per sublayer and the height of each sublayer (*h*). A specific quantity, called ε, is also defined as *v_cell_ r - r.* The variable ε will be used during the actual cell placement phase.

2. **cells placement in 2D (planar) for each sublayer**; the first part of the placement algorithm place the cells over the 2D plane, not taking into account the thickness of the sublayer. This part of placement procedure can be described as follows:
   - **i.** Randomly select a point *C* over the plane - place the first cell (cell 1);
   - **ii.** Draw a circle with center *C* and radius *r*; sample a certain number of Possible Points (*PP,* default is 50) on the circle and linearly project them: their distance *d* from *C* will be equal to $2r \pm k \cdot \varepsilon$. *k* is sampled from a random uniform distribution ranging from 0.75 to 1.25.
   - **iii.** If only cell #1 has been placed: randomly select a *PP* and place cell #2 according to **ii**

iv. If a number of cells *n*> 1 has been already placed, then for each *PP* calculate *d* from *n*-1 cells; if *d* always <= 2*r*, place cell *n*+1. If *d* never <= 2*r* (i.e., no *PP* is a good candidate for the next cell N), go back to cell #1, select a different *PP* from that used for placing cell #2 and check if the next cell can be placed there. If no *PP* are eligible as next cell, go to cell 2, 3,..., *n*-1 until a good *PP* is found. If it is not possible to place the next cell, break the loop and go to the next sublayer. Otherwise, continue the cell placement procedure until all the cells are positioned.

3. **add the third dimension**; a range of possible vertical coordinates for the center of the somata is randomly sampled from a uniform distribution with fixed boundaries: given the heights of sublayers, *h*, and the aforementioned radius of the cell type, *r*, upper boundary is equal to *h*-*r* while lower boundary equals to *r*. Lastly, sublayers are stacked on top of each other to rebuild the entire layer

See the figure below for an illustrated example of the placement algorithm; see the file placementSC.avi for a movie of cell placement of stellate cells.

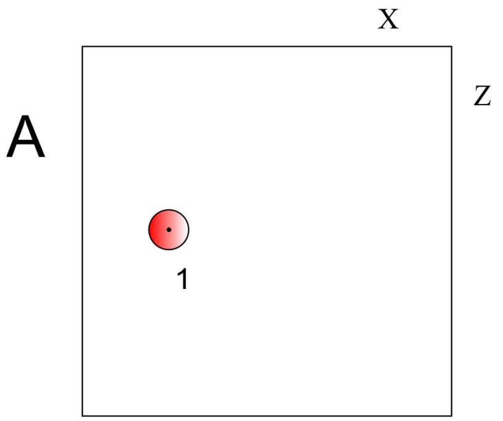

Randomly choose a point in the 2D plane.
This will be the center of the cell soma.
The circle radius (***r***) corresponds to the radius of the cell type soma

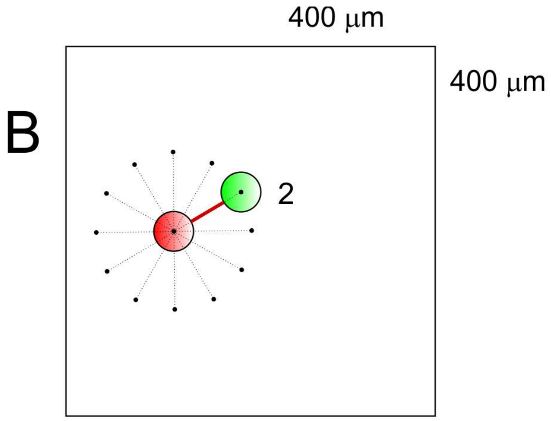

Sample some points on circunference, project them onto the plane. Distance (***d***) between projected points and the centre of first cell = 2**r** + $\kappa\varepsilon$., with ε = compound sphere radius - **r** , κ = random between 0.75 and 1.25. Randomly select one point and place the second cell (green)

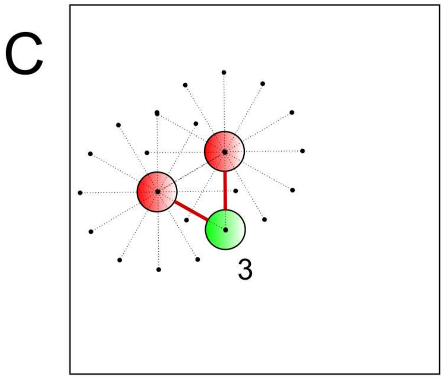

From cell 2, apply the same algorithm to find possible points for cell 3.
For each point, calculate its distance from cell 1 too
(i.e., from all previously placed cells).
If ***d*** always >= 2***r*** + $\kappa\varepsilon$ then place cell 3

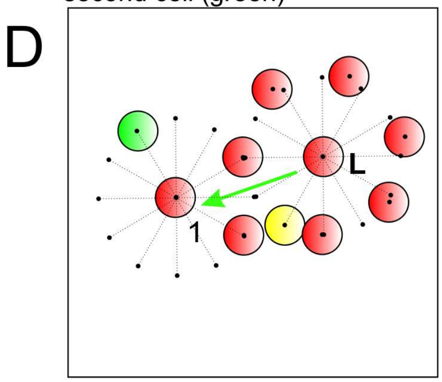

Possible points projected from the last placed cell **L** are too close to at least one other cell
(***d*** < 2***r*** + ε; see the yellow circle).
Go back to the first cell (green arrow) and search for other possible points (green circle).
If no points are available, go to cell 2, 3 ... n.
If no points are available from any cell, the algorithm ends.

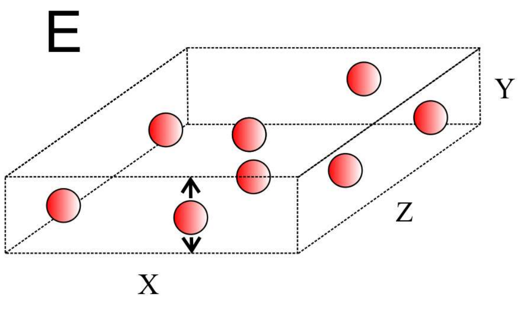

Once a sublayer is filled, each cell can be placed along the third dimension (Y axis) by randomly 'lifting' the cell itself within the range of sublayer height (i.e., size of Y axis)

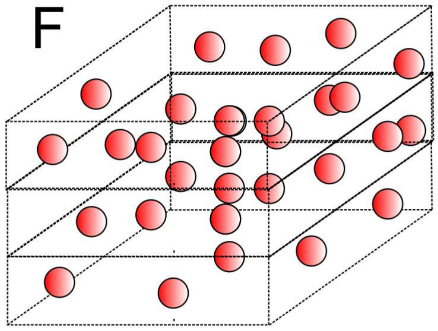

Apply the same algorithm (A-E) for all sublayers; stack each sublayer on the top of the previous one to build the entire layer

Connectivity Rules

1. Glom-GrC: excitatory

Granule cells can have no more than 4 dendrites, each with a maximum length of 40µm, developing freely in all directions (Dieudonne, 1998; D'Angelo *et al.*, 2013). Therefore, a connection can be generated only if the distance between the GrC soma and the Glom center is less than GrC dendrites length. Each dendrite can have only a single connection with a glomerulus, hence if the number of glomeruli that satisfy the condition is more than the number of dendrites, the four closer ones are chosen (Solinas *et al.*, 2010).

2. Glom-GoC: excitatory

GoC basolateral dendrites occupy a volume that resembles a semi-sphere under the GoC soma, with an arborization of 50µm radius (Kanichay and Silver, 2008). Each glomerulus located into this dendritic GoC volume connects exclusively to that GoC.

3. GoC-Glom: inhibitory

GoC axon develops from the soma in a spiral shape in the sagittal plane, that can reach an extension of 150 µm on both x- and y-axes, while being very narrow on the z-axis (Solinas *et al.*, 2010). Each axon forms a maximum of 40 connections with as many different glomeruli (D'Angelo *et al.*, 2013). Therefore, if the number of post-synaptic cells that fall under this volume exceeds such physiological divergence value, the nearest 40 ones are connected (Table 2). Then, the glomeruli inhibited by GoCs inhibit in turn all GrCs connected to those Gloms (from connection type 1).

4. GoC-GoC: inhibitory

This type of connectivity occurs between the axon of the pre-synaptic GoC and the dendrites of the post-synaptic GoC; therefore only geometrical constraints have been set in this case, consequently choosing all post-synaptic GoCs that satisfy the condition (Hull and Regehr, 2012).

5. aa-GoC: excitatory

Golgi cells basolateral and apical dendrites form a sphere around the soma, with an arborization of 50µm radius (Kanichay and Silver, 2008), with the latter going up to reach the molecular layer. Hence, ascending axons of granules form connections if they cross that volume. In addition, even GrC cells are considered whose soma is situated into a virtual cylindrical volume where the base is a circle centered on the GoC soma and with radius of 50µm, while the height is equal to that of the corresponding potential post-synaptic GoC y coordinate. Each ascending axon can establish only one connection with a GoC, while a single Golgi cell can have no more than 400 connections, selected with a probability-based rule.

6. PF-GoC: excitatory

GoC apical dendrites connect also with the parallel fibers originated from the same GrC that form connections with their ascending axons, together with other PF randomly chosen that cross the area formed by the apical dendrites, with the goal of reaching a total convergence value of 1600(Solinas *et al.*, 2010).

7& 8. SC-SC and BC-BC: inhibitory

The dendritic tree of both stellate and basket cells is almost like a circle on the sagittal plane. Each molecular interneuron forms a connection with other four different cells of the same type, which are selected using a probability rule, among the potential post-synaptic neurons that fall into the pre-synaptic dendritic tree (Lennon *et al.*, 2014).

9 & 10. pf-SC and pf-BC: excitatory

In general, PF *x* and *z* coordinates are the same of the originating granule cell, while their height is the same as that of the corresponding ascending axon. Instead, stellate and basket cells dendritic tree is a circle centered on the soma and with a radius of 15μm in the sagittal plane. In this case, the geometry is the only constraint to be set as connectivity rule (Jorntell *et al.*, 2010).

11 & 12. SC-PC and BC-PC: inhibitory

Purkinje dendritic tree is almost planar on the sagittal plane, therefore its area is a rectangle of entire molecular layer height and base width of 130μm centered on the Purkinje soma. Therefore, 20 stellate or basket cells selected with a Poisson probability rule between the ones that fall into the area described, make a connection (Lennon *et al.*, 2014).

13. aa-PC: excitatory

Since Purkinje dendritic tree extends for the entire height of the molecular layer, only *x* and *z* coordinates matter in terms of geometrical constraints. In particular, the considered area is a parallelepiped with a very narrow *z* width: each ascending axon that satisfies the geometrical condition can connect with a single PC (Huang *et al.*, 2006).

14. pf-PC: excitatory

In this paper, only parallel fibers originated from positioned GrC are considered and, since their mean length is of about 2mm (Huang *et al.*, 2006), PF are assumed to extend for the entire simulation volume (here, no more than 400μm). In this way, it is sufficient to check whether their x coordinate falls into the dendritic tree.

15. PC-DCN: inhibitory

First, it is necessary to determine the dendritic tree of each DCN cell: since its dimensions exceed those of the simulation volume, a plane with a random orientation angle with respect to the canonical three planes represents it (Sultan and Heck, 2003). After that, the connectivity is performed(Person and Raman, 2012): each PC (pre-synaptic neuron) is linked randomly to 4-5 different DCN cells (coincident with the divergence). The number of glutamatergic DCN projection large neurons within the reconstructed volume is very limited. It has to be noted that here it is not significant to calculate the minimum distance between PC soma and DCN dendritic tree, due to the very high distance between different layers. However, with a wider volume, it will be possible to implement a more articulated connectivity rule, which resembles literature.

16. Glom-DCN: excitatory

Mossy fiber collaterals strengthen cerebellar output by connecting with glutamatergic DCN neurons; more specifically, at present 147 glomeruli (as per convergence value, Boele *et al.*, 2013) are randomly chosen to form connections with this type of DCN neurons.

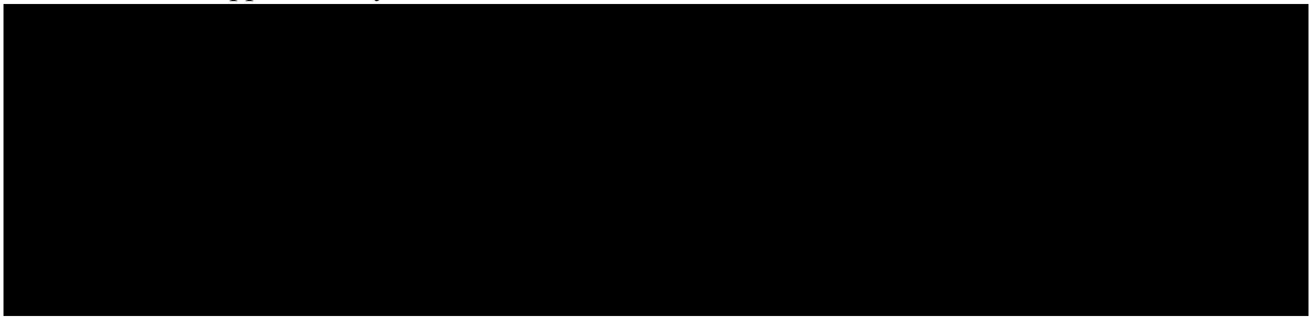

Figure S1

The panel A shows the somata of all the stellate cells placed into the sample volume (within the upper half of the molecular layer). The red cellis pre-synaptic; the light blue and the blue cells are the potential post-synaptic cells identified by intersection of axonal and dendritic fields; the blue cells are those effectively connected (given the divergence ratio 1:4). The panel B shows schematically the geometry of the pre-synaptic axonal field (red cylinder) and of the post-synaptic dendritic field (blue cylinder). Note that the axonal cylinder elongates along the transverse axis, while the dendritic cylinder is quite thin with the basis on the sagittal plane.

References

Boele, H. J., Koekkoek, S. K. E., De Zeeuw, C. I. and Ruigrok, T. J. H. (2013) 'Axonal Sprouting and Formation of Terminals in the Adult Cerebellum during Associative Motor Learning', *Journal of Neuroscience*, 33(45), pp. 17897-17907.

D'Angelo, E., Solinas, S., Garrido, J., Casellato, C., Pedrocchi, A., Mapelli, J., Gandolfi, D. and Prestori, F. (2013) 'Realistic modeling of neurons and networks: towards brain simulation', *Funct Neurol*, 28(3), pp. 153-66.

Dieudonne, S. (1998) 'Submillisecond kinetics and low efficacy of parallel fibre Golgi cell synaptic currents in the rat cerebellum', *Journal of Physiology-London*, 510(3), pp. 845-866.

Huang, C. M., Wang, L. and Huang, R. H. (2006) 'Cerebellar granule cell: ascending axon and parallel fiber', *European Journal of Neuroscience*, 23(7), pp. 1731-1737.

Hull, C. and Regehr, W. G. (2012) 'Identification of an Inhibitory Circuit that Regulates Cerebellar Golgi Cell Activity', *Neuron*, 73(1), pp. 149-158.

Jorntell, H., Bengtsson, F., Schonewille, M. and De Zeeuw, C. I. (2010) 'Cerebellar molecular layer interneurons - computational properties and roles in learning', *Trends in Neurosciences*, 33(11), pp. 524-532.

Kanichay, R. T. and Silver, R. A. (2008) 'Synaptic and cellular properties of the feedforward inhibitory circuit within the input layer of the cerebellar cortex', *J Neurosci*, 28(36), pp. 8955-67.

Lennon, W., Hecht-Nielsen, R. and Yamazaki, T. (2014) 'A spiking network model of cerebellar Purkinje cells and molecular layer interneurons exhibiting irregular firing', *Frontiers in Computational Neuroscience*, 8, pp. 1-10.

Person, A. L. and Raman, I. M. (2012) 'Synchrony and neural coding in cerebellar circuits', *Front Neural Circuits*, 6, p. 97.

Solinas, S., Nieus, T. and D'Angelo, E. (2010) 'A realistic large-scale model of the cerebellum granular layer predicts circuit spatio-temporal filtering properties', *Front Cell Neurosci*, 4, p. 12.

Sultan, F. and Heck, D. (2003) 'Detection of sequences in the cerebellar cortex: numerical estimate of the possible number of tidal-wave inducing sequences represented', *Journal of Physiology-Paris*, 97(4-6), pp. 591-600.
